# Normative influence in a time of distrust and polarization: how perceived social norms predict COVID-19 vaccination intentions among Black Americans

**DOI:** 10.1007/s10865-025-00578-7

**Published:** 2025-06-09

**Authors:** Tong Lin, Yuan Wang, Kathryn Thier, Xiaoli Nan

**Affiliations:** 1 Department of Communication, University of Maryland, College Park, MD USA; 2 Wee Kim Wee School of Communication and Information, Nanyang Technological University, Singapore, Singapore

**Keywords:** Social norms, COVID-19 vaccination, Health disparities, Health promotion

## Abstract

This study examined the impact of social norms—descriptive, injunctive, and subjective norms—on COVID-19 vaccination intentions among Black Americans. It also investigated how Black Americans affiliated with Democrats and Republicans perceived norms within their groups and how such perceptions influenced their COVID-19 vaccination intentions. We conducted an online national survey with Black Americans (*N* = 1497) between February and March 2021. Results showed that all three types of norms were significantly and positively associated with vaccination intentions, with subjective norms exerting the strongest effect. Additionally, higher subjective norms amplified the positive relationship between descriptive norms and vaccination intentions. Republicans were influenced by both in-group and out-group norms, while Democrats were primarily guided by in-group norms, underscoring the potential of bipartisan messaging to boost vaccine uptake. These findings highlight how social norms impact COVID-19 vaccination intentions and reveal that individuals with differing party affiliations are influenced differently based on their perceived identification with reference groups. The study provides insights for designing targeted interventions to improve vaccine uptake among Black Americans and for developing broader public health messaging strategies. It contributes to the literature by revealing how the interplay of descriptive, injunctive, and subjective norms shapes vaccination intentions and by offering a novel perspective on the differentiated effects of in-group and out-group norms based on partisan identity.

## Introduction

The COVID-19 pandemic has had a devastating societal impact, claiming numerous lives and undermining individuals’ trust in public institutions (Goldstein & Wiedemann, 2021; Han et al., 2021). The elevated distrust is particularly detrimental among marginalized communities, such as Black Americans, who tend to be distrustful of the medical establishment due to historical injustice and mistreatment (Rogers et al., 2022; Thompson et al., 2021). Amid growing distrust in institutions, people increasingly rely on others in their communities to guide critical decisions, such as responding to a pandemic. The COVID-19 pandemic has also exacerbated political polarization, with Democrats and Republicans exhibiting starkly divergent levels of protective behaviors, including whether to get vaccinated (Druckman et al., 2021; Jiang et al., 2021). With increasing distrust and polarization, it is crucial to understand the dynamics of normative influence in pandemic decision-making. Normative influence refers to the impact that perceived norms – the implicit or explicit rules that a group uses to determine appropriate and inappropriate values, beliefs, attitudes, and behaviors – have on an individual’s behavior (Cialdini et al., 1991). In the context of health behaviors, such as the decision to receive a COVID-19 vaccine, these social norms can play a significant role (Graupensperger et al., 2021). In this paper, we examine how perceived social norms could predict COVID-19 vaccination intentions among Black Americans. Additionally, we investigate how Black Americans identifying with Democratic or Republican political parties might be susceptible to different normative influences generally and when associated with reference groups marked by Democratic and Republican political ideologies. Our study provides insight for health communicators in designing tailored interventions to enhance Black Americans’ COVID-19 vaccine uptake. Our findings also hold implications for designing effective COVID-19 public health messages more broadly for the general population.

### Perceived norms and COVID-19 vaccination intentions

Within health communication and social psychology, scholars have explored social norms’ role in influencing individuals’ behavior (Chen et al., 2023). On the one hand, studies have demonstrated the explanatory and predictive value of social norms under different health conditions and social settings (e.g., Rhodes et al., 2020; Rimal & Lapinski, 2015). On the other hand, scholars posited the limitations of applying the concept of social norms to explain human behavior if the behavior is transmitted through socialization or under the influence of culture and policies (Tillman et al., 2019). Since the effects of social norms are distinct from one study to another, research attention has turned to whether different types of social norms affect behavior in different scenarios.

### Descriptive norms

Social norms are conceptualized as guidelines and rules of behavior that occur at the formal and informal levels (Bicchieri et al., 2011). As one of the informal norms, descriptive norms address the “folkways” that represent the group’s characteristics, which guide group members’ behaviors in accordance with the informal customs (Chung & Rimal, 2016). This type of norm characterizes the effect of imitation through people’s perceptions of what significant others themselves do (Anderson & Dunning, 2014). As indicated in social norms theory, people may be influenced by incorrect perceptions of how others in their social groups behave (Johnson, 2012). The essence of descriptive norms, as discussed by previous studies such as Kallgren et al. (2000) and Shulman et al. (2017), is not to challenge the truthfulness of perceptions. Instead, their purpose is to offer social insights by examining commonly held perceptions of others’ actions in relation to a specific behavior. Violating descriptive norms tends to be free from consequences because one’s personal behavior may not always align with these perceptions.

Research has extensively documented the influence of descriptive norms on behavioral intentions. Scholars have posited that descriptive norms serve as cognitive shortcuts in decision-making processes (Cialdini et al., 1990; Xiao & Borah, 2020). This theoretical perspective suggests that individuals, when exposed to prevalent behaviors within their social milieu, are more inclined to adopt similar behaviors due to a sense of social proof (Rivis & Sheeran, 2003). Empirical studies have supported this notion by revealing positive associations between descriptive norms and various health-related behaviors. For instance, researchers have demonstrated that descriptive norms correlate positively with household intentions regarding PM_2.5_ (air pollution) reduction behavior (Shi et al., 2017), indoor tanning practices (Carcioppolo et al., 2017), and the consumption of functional foods^1^ (Nystrand & Olsen, 2020).

Within the context of vaccination behavior, an extensive body of literature underscores the role of perceiving vaccination as a social norm in driving vaccine acceptance (Dubé et al., 2013). Notably, numerous studies have elucidated the constructive impact of descriptive norms on vaccination promotion campaigns (e.g., Visser et al., 2018; Xiao & Borah, 2020) and vaccination intentions (e.g., Moehring et al., 2021; Wang et al., 2017). Moreover, recent research on the COVID-19 pandemic has provided compelling evidence of the significant positive effect of descriptive norms on vaccination intention among Black Americans (e.g., Bogart et al., 2022). These findings collectively underscore that individuals are more likely to express confidence in the effectiveness of vaccination and opt for immunization when they perceive that others within their community share similar vaccination intentions.

### Injunctive norms

Another type of informal norm that is commonly perceived is the injunctive norm. Injunctive norms refer to the perceptions of “what others think should be done” (Chung & Rimal, 2016, p. 6) or “what should or ought to be done with respect to performing a given behavior” (Fishbein & Ajzen, 2010, p. 131). More favorable injunctive norms tend to guide behaviors because individuals have a higher desire to be part of a group formed by shared thoughts and values. The underlying motivation of perceived injunctive norms is to gain social approval or acceptance (Kollerová et al., 2018).

In contrast to descriptive norms, the violation of injunctive norms often engenders distinct social dynamics (Chen & Hong, 2015). Such violations may lead to increased engagement in behaviors that are not socially acceptable or are considered unethical (e.g., Cardella et al., 2019; Diekmann et al., 2015). For example, Keizer et al.’s studies (2008; 2011) have demonstrated that individuals are more inclined to transgress regulations against graffiti spraying in public settings when they perceive others doing the same, even when anti-graffiti signage is present. However, it is noteworthy that injunctive norms can be positively framed and garner favorable responses when the behavior in question is beneficial to society at large. Previous research has underscored the significance of injunctive norms as potent predictors of various health-related behaviors. These behaviors encompass domains such as alcohol consumption (Lewis et al., 2015) and altruistic actions like organ donation (Habib et al., 2021). Moreover, empirical evidence suggests that injunctive norms exert a considerable influence on vaccination intentions, as exemplified by studies examining vaccination behavior among rural adolescents in the context of influenza vaccines (Painter et al., 2010) and among Latina and Non-Latina White Women with regard to human papillomavirus (HPV) vaccines (Reimer et al., 2013).

Limited research has explored the impact of injunctive norms on COVID-19 vaccine adoption within the Black American population. Within this context, some researchers have yielded intriguing findings, revealing that normative pressures significantly influence vaccination intentions among older adults, with no apparent variations observed across racial demographics (Xiao & Borah, 2020). Drawing insights from the broader vaccination literature, it becomes evident that favorable injunctive norms hold the potential to foster compliance by underscoring collective responsibilities in adhering to guidelines encompassing mask-wearing, social distancing, and vaccination (e.g., Baeza-Rivera et al., 2021). Conversely, when individuals harbor uncertainties regarding whether others endorse the notion of vaccination, they may exhibit skepticism or reluctance toward inoculation efforts. This underscores the intricate interplay between injunctive norms and vaccination decision-making, particularly in the context of COVID-19, where collective action plays a pivotal role in curbing the spread of the virus.

### Subjective norms

Closely aligned with injunctive norms, the concept of subjective norms pertains to the perceived social pressure that an individual experiences from significant individuals in their social sphere regarding a specific behavior (Ajzen, 1991). Some scholars have proposed an extension of the scope of subjective norms to encompass normative beliefs related to the behaviors of these significant others, effectively merging the concept with injunctive norms (Fishbein & Ajzen, 2010). Conversely, others have emphasized a distinction between the two, asserting that subjective norms primarily center on an individual’s perception of the expectations regarding what they can or should do, separate from the behavior of others (Shin & Hancer, 2016). In essence, subjective norms are characterized by the heightened perception of expectations originating from significant individuals, including friends and family. These perceived expectations often serve as motivational factors that encourage individuals to engage in a particular behavior. Numerous prior studies have underscored the paramount importance of subjective norms in various contexts, affirming their significant influence on human behavior. In the context of COVID-19, positive subjective norms may lead to a greater willingness to get the COVID-19 vaccine if individuals perceive higher social pressure or expectations from their significant others (e.g., Guidry et al., 2021; Huynh et al., 2021). The relationship between subjective norms and the intent to vaccinate is particularly potent among Black Americans; several studies have underscored the importance of emphasizing the prosocial benefits of vaccination (e.g., Bogart et al., 2022; Guidry et al., 2021). From these insights, we hypothesize the following:

#### H1

*Higher perceived descriptive norms are associated with greater COVID-19 vaccination intentions.*

#### H2

*Higher perceived injunctive norms are associated with greater COVID-19 vaccination intentions.*

#### H3

*Higher perceived subjective norms are associated with greater COVID-19 vaccination intentions.*

### Differential effects of perceived norms

Although prior research has underscored the significant influence of social norms on behavior, it remains uncertain which type of norm is more impactful. Theories such as the Theory of Normative Social Behavior (TNSB; Real & Rimal, 2007; Rimal & Real, 2005) and the Focus Theory of Normative Conduct (Cialdini, 2003) differentiate between injunctive and descriptive norms, suggesting varying levels of impact based on the topic and context. Earlier discussions have noted that the sources of motivation differ among subjective, injunctive, and descriptive norms (Rivis & Sheeran, 2003). Further studies reveal that these norms not only have different predictive powers but also distinct influences on outcomes. For instance, Park and Smith (2007) examined five types of social norms (subjective, personal, descriptive, injunctive, and societal descriptive and injunctive) on the intent to register as an organ donor and discussions about organ donation with family, finding unique effects for each norm type on these behaviors. Another study by Mollen et al. (2013) showed that while injunctive norms significantly influenced intervention behaviors in alcohol consumption contexts, descriptive norms did not. This suggests that while injunctive norms are more resistant to change compared to other types of norms, the changes that do occur tend to have a more enduring impact. The effects of social norms can also be different in different target populations. Graupensperger et al.’s (2021) study showed that college students had greater intentions to get a COVID-19 vaccine than an influenza vaccine. However, on average, their student participants thought other young adults were less likely to be vaccinated (i.e., lower descriptive norms) and would not believe vaccination was as important (i.e., lower injunctive norms). Such perceptions of social norms were positively correlated with their vaccination intentions. Given the existing evidence demonstrating lower vaccine completion rates among Black Americans (e.g., Galbraith et al., 2016; Moore et al., 2021), our focus lies in understanding which specific types of perceived norms exert the most significant influence on COVID-19 vaccination intentions among Black American adults.

Additionally, as proposed by the TNSB, injunctive norms moderate the relationship between descriptive norms and behaviors, and the effect should become stronger as injunctive norms become stronger (Rimal, 2008). However, recent health communication studies analyzing the moderating role of injunctive norms found contradicting results. For instance, Yang (2017) found insignificant interaction effects between descriptive and injunctive norms on college students’ intentions to consume alcohol. Jain et al.’s (2018) work has also revealed that injunctive norms did not moderate the relationship between descriptive norms and condom use. Hence, we also explore whether there are significant interactions between descriptive, injunctive, and social norms on the intention to receive the COVID-19 vaccine. We ask the following questions:

#### *RQ1*

*Which type of perceived norms (descriptive, injunctive, subjective) is most strongly associated with COVID-19 vaccination intentions?*

#### *RQ2*

*Are there any interaction effects among descriptive, injunctive, and subjective norms on the intentions to obtain the COVID-19 vaccine?*

### Normative influence and reference groups

The process in which individuals form their beliefs about scientific consensus is influenced by their cultural cognitions (Kahan et al., 2011), while disagreement on science and health issues is highly polarized across social groups (e.g., Nisbet & Markowitz, 2014). Kerr et al.’s (2021) study revealed that political polarization about COVID-19 exists among the U.S. public, with liberals reporting engaging in a higher number of COVID-19 health preventive behaviors than conservatives. Individuals form part of their self-definition from their group membership in which their perceptions, attitudes, beliefs, and behaviors are in line with the norms of the group (Islam, 2014). The values shared within groups provide the intellectual and moral justification for decision-making (Hornsey & Fielding, 2017). Drawing from the self-cognitive theory (Bandura, 2009), individuals are understood to affiliate with a particular political party as a means to address their psychological needs (Vraga, 2015).

From a personality trait perspective, individuals with high scores in social dominance orientation (SDO) prioritize the interests of their in-group and endorse dominance and inequality across groups, regardless of their in-group’s position in the power hierarchy (Pratto et al., 1994). Conversely, those who score high on right-wing authoritarianism (RWA) tend to defer to authority figures, resist groups that challenge traditional norms, and act aggressively toward those opposing such authorities (Altemeyer, 2004). Past research identifies RWA and SDO as predictors of science rejection across various topics (Kerr & Wilson, 2021). Additionally, normative perceptions are significantly influenced by political affiliations, as individuals often rely on shared group values and information to guide decision-making (Burchell et al., 2013). Scholarly studies consistently highlight the pivotal role of group identification in shaping beliefs, demonstrating that individuals frequently adopt views aligning with their in-groups, such as those of members within the same political party (Shi et al., 2022).

Recent studies examining the normative influence on environmental and health behaviors have pointed out that, despite group identification, norm specificity (i.e., normative reference group) is indissociable when discussing the impact of social norms (Mertens & Schultz, 2021). Norm specificity focuses on the degree of proximity a person perceives as related to a referent group. A referent group is a social group used as a behavioral comparison (Borsari & Carey, 2003) rather than a group that the individual identifies with. Studies have shown that individuals are more likely to be persuaded to adopt a behavior when considering the social norms in a reference group with a higher degree of proximity (Lac & Donaldson, 2018). In Cho’s (2006) study, the impact of friends’ norms on binge drinking was found to be more significant than that of the campus’s norms, which were relatively less influential due to their distant proximity. Similar trends were also evidenced by Zhuang (2022), demonstrating that perceived norms among various reference groups, including Black Americans, had varying effects on vaccination intentions. These studies have indicated the central elements when evaluating specificity, such as spatial proximity (e.g., neighbors), genetic proximity (e.g., family members), and social proximity (e.g., same race/ethnicity). However, few studies have investigated social norms about vaccination within the reference groups of political party affiliations.

People’s affiliations with different political parties might cause variations in how they are influenced by social norms, given their degree of identification with a reference group. This, in turn, can impact their intentions regarding COVID-19 vaccination (Bavel et al., 2020). While group identification and the specificity of norms are distinct concepts, it is plausible to suggest that stronger identification with a reference group magnifies the relationship between social norms and health behaviors (e.g., Fielding et al., 2020). Previous research on party affiliation identification has shown that individuals respond more favorably to policies supported by their in-group members compared to those supported by out-group members (Cohen, 2003; Fielding et al., 2020). This suggests that individuals might be swayed to either accept or decline vaccination based on the attitudes and actions of those who share their political party affiliation.

In this study, our aim was to discern how Black Americans affiliated with distinct political parties perceive norms within their groups and how such perceptions influence their COVID-19 vaccination intentions. We focused on both descriptive and injunctive norms as perceived within the two main political groups: Democrats and Republicans. We pose the following research question:

#### RQ3

How are perceived descriptive and injunctive norms associated with COVID-19 vaccination intentions among Democratic and Republican Black Americans?

## Method

### Participants and procedure

We conducted an online survey and recruited participants through Qualtrics’ online panels between February 10 and March 11, 2021. Eligible participants included people 18 or older, who self-identified as Black Americans, and had not received a COVID-19 vaccine at the time. Nationally representative quotas on gender, age, and education were used to ensure a diverse sample. Participants read an introduction page of the survey and signed a consent form online. The study protocol was approved by the University of Maryland Institutional Review Board.

A total of 1,497 eligible participants completed the online survey. Table 1 shows the demographic composition of the samples. Participants ranged from 18 to 81 years old (*M* = 40.62, *SD* = 14.07). The sample included 43.9% (*n* = 657) males, 52.4% (*n* = 784) females, 1.9% (*n* = 28) non-binary individuals, and 1.9% (*n* = 28) individuals who self-identified as other gender. Among the participants, 5.2% (*n* = 78) had less than high school education, 35.7% (*n* = 534) were high school graduates, 28.1% (*n* = 420) had some college, 10.6% (*n* = 158) had an associate degree, 15% (*n* = 224) had a college degree, and 5.5% (*n* = 83) had a postgraduate degree. Participants’ ideology ranged from very liberal (*n* = 137, 9.2%), liberal (*n* = 281, 18.8%), moderate (*n* = 809, 54.0%), conservative (*n* = 170, 11.4%), to very conservative (*n* = 100, 6.7%). Participants consisted of 61.5% (*n* = 920) Democrats, 5.6% (*n* = 84) Republicans, 24.7% (*n* = 370) Independents, and 8.2% (*n* = 123) individuals with other political party affiliations.

**Table 1:** The demographic composition of the sample (*N* = 1,497)

Variable	Categories	Frequency	Percent (%)
Gender	Male	657	43.9
	Female	784	52.4
	Non-binary	28	1.9
	Other	28	1.9
Age	Under 23	197	13.2
	23–35	387	25.9
	36–55	612	40.9
	56 and above	301	20.1
Education	Less than high school	78	5.2
	High school graduate	534	35.7
	Some college	420	28.1
	Associate’s degree	158	10.6
	College Graduate	224	15.0
	Post-graduate	83	5.5
Political Ideology	Very liberal	137	9.2
	Liberal	281	18.8
	Moderate	809	54.0
	Conservative	170	11.4
	Very conservative	100	6.7

### Measures

**Intention to vaccinate against COVID-19*.*** Intention to vaccinate against COVID-19 was measured with the following item asking: “If a COVID-19 vaccine becomes available to you free of cost, would you get it?” Responses were indicated on a six-point scale (1 = *Definitely no*, 2 = *Probably no*, 3 = *Leaning toward no*, 4 = *Leaning toward yes*, 5 = *Probably yes*, 6 = *Definitely yes*) (*M* = 3.58, *SD* = 1.85).

**Descriptive norms*.*** Participants were asked to answer five questions: “Think about 1) all people in the U.S, 2) all people of your race, 3) all people of your age, 4) all people who identify as Democrats, and 5) all people who identify as Republicans, about how many of them do you think will get a COVID-19 vaccine?” Participants indicated their perceived descriptive norms among the five reference groups on a five-point scale (1 = *Few (0 to 20%), 2* = *Less than half (21% to 40%), 3* = *Around half (41% to 60%), 4* = *More than half but not all (61% to 80%), 5* = *Most or nearly all (81% to 100%)*)*.* The five items were averaged to form an index for descriptive norms (*M* = 3.07, *SD* = 0.81, Cronbach’s ⍺ = 0.81).

**Injunctive norms**. Five items assessed injunctive norms on a seven-point scale (1 = *Not at all*, 7 = *Very much so*): “Think about 1) all people in the U.S, 2) all people of your race, 3) all people of your age, 4) all people who identify as Democrats, and 5) all people who identify as Republicans, do you believe they think it is a good idea to get a COVID-19 vaccine?” The five items were averaged to form an index for injunctive norms (*M* = 4.38, *SD* = 1.39, Cronbach’s ⍺ = 0.86).

**Subjective norms*.*** Two items assessed subjective norms on a seven-point scale (1 = *Not at all*, 7 = *Very much so*): “Think about all people who are close to you, 1) do they want you to get a COVID-19 vaccine, and 2) do they think it is important for you to get a COVID-19 vaccine?” The two items were averaged to indicate subjective norms (*M* = 4.26, *SD* = 1.91, Cronbach’s ⍺ = 0.91).

**Control variables.** Participants reported their age, gender, education, political ideology, and political party affiliation. Political ideology refers to participants’ liberal–conservative orientation, whereas party affiliation indicates whether they identify as Democrat, Republican, Independent, or as individuals affiliated with another political party. Gender was dummy-coded into three variables: female (vs. male), non-binary (vs. male), and other (vs. male).

### Data analysis

We conducted a hierarchical linear regression analysis to examine the influence of perceived norms on individuals’ intentions to obtain the COVID-19 vaccine, after controlling for demographic variables, including age, gender, education, and political ideology. We performed moderation analyses using PROCESS (Hayes, 2012) to examine the interaction effects among perceived descriptive, injunctive, and subjective norms on COVID-19 vaccination intentions. To address RQ 3, we conducted two hierarchical linear regression analyses to explore the impact of individual types of perceived norms on vaccination intentions among individuals of different party affiliations (Democrats vs. Republicans). All analyses were done using SPSS 28.0. All statistical tests were evaluated using a significance level of *p* < 0.05.

## Results

### Descriptive statistics

Overall, participants held relatively high perceived injunctive norms (*M* = 4.38, *SD* = 1.39) and subjective norms (*M* = 4.26, *SD* = 1.91). In other words, participants by and large agreed that others approved of COVID-19 vaccination. Perceived descriptive norms showed a mean score of 3.07 (*SD* = 0.81), suggesting that participants believed that about half of the individuals in other reference groups had been vaccinated against COVID-19. Intentions to get COVID-19 vaccination were moderate (*M* = 3.58, *SD* = 1.85). The zero-order correlations among the three types of perceived norms and vaccination intentions were all statistically significant (all *p*s < 0.01) with correlation coefficients ranging from 0.40 to 0.71.

### Hypotheses testing and research questions

We tested all hypotheses using hierarchical linear regression. Assumptions of normality, homoscedasticity, linearity, independence of errors, and absence of outliers were checked and met before performing the analyses. Control variables included key demographic measures such as gender, age, and education, in addition to political ideology. Gender was dummy-coded into three variables: female, non-binary, and other gender. In Table 2, the first model (Block 1) included the control variables: age, gender, education level, and political ideology. The second model (Block 2) was a full model by adding the perceived norms constructs. Results showed that adding the perceived norms constructs significantly increased the amount of variance explained for vaccination intentions (Δ*R*^*2*^ = 0.40, *p* < 0.001). The full model explained 45.7% of the variance in vaccination intentions. Consistent with H1 through H3, perceived descriptive norms (*b* = 0.25, *p* < 0.001), injunctive norms (*b* = 0.22, *p* < 0.001), and subjective norms (*b* = 0.46, *p* < 0.001) were all significantly and positively associated with COVID-19 vaccination intentions. Thus, H1, H2, and H3 were supported.

**Table 2:** Hierarchical regression results predicting COVID-19 vaccination intentions

Variable	*b*	*b* 95% CI [LL, UL]	*b (SE)*	*ß*	*t*	*R*^*2*^	Difference
*Block 1*						0.058^***^	
Constant	2.93^***^	[2.49, 3.37]	0.22				
Age	0.03^***^	[0.02, 0.03]	0.00	0.20			
Female	− 0.24^*^	[− 0.44, − 0.05]	0.10	− 0.07			
Non-binary	− 0.21	[− 0.90, 0.47]	0.35	− 0.02			
Other Gender	0.13	[− 0.55, 0.82]	0.35	0.01			
Education Level	0.08	[0.01, 0.15]	0.04	0.06			
Political Ideology	− 0.20^***^	[− 0.30, − 0.10]	0.05	− 0.10			
*Block 2*							
Constant	0.12	[− 0.29, 0.53]	0.21			0.457^***^	*∆R*^*2*^ = 0.40^***^
Age	0.01	[− 0.00, 0.01]	0.00	0.04			
Female	− 0.06	[− 0.20, 0.09]	0.08	− 0.02			
Non-binary	-0.01	[− 0.53, 0.51]	0.27	− 0.00			
Other gender	0.14	[− 0.38, 0.66]	0.27	0.01			
Education level	0.01	[− 0.05, 0.06]	0.03	0.01			
Political ideology	− 0.13^***^	[− 0.21, − 0.06]	0.04	− 0.07			
Descriptive norms	0.25^***^	[0.15, 0.35]	0.05	0.11			
Injunctive norms	0.22^***^	[0.14, 0.29]	0.04	0.16			
Subjective norms	0.46^***^	[0.40, 0.51]	0.03	0.47			
*Block 3*							
Constant	0.39	[− 0.40, 1.17]	0.40		0.97	0.462^***^	*∆R*^*2*^ = 0.004^***^
Age	0.01	[− 0.00, 0.01]	0.00	− 0.01	1.88		
Female	− 0.05	[− 0.20, 0.10]	0.07	0.00	− 0.70		
Non-binary	0.01	[− 0.51, 0.53]	0.26	0.01	0.05		
Other gender	0.13	[− 0.39, 0.65]	0.26	0.04	0.48		
Education level	0.01	[− 0.05, 0.06]	0.03	0.01	0.28		
Political ideology	− 0.13^***^	[-0.20, − 0.06]	0.04	− 0.07	− 3.49		
Descriptive norm	0.13	[− 0.15, 0.41]	0.14	0.06	0.93		
injunctive norm	0.52^***^	[0.24, 0.80]	0.14	0.40	3.67		
Subjective norm	0.08	[− 0.16, 0.32]	0.12	0.09	0.68		
Descriptive norm X Injunctive norm	− 0.10^*^	[− 0.18, − 0.01]	0.04	− 0.36	− 2.18		
Descriptive norm X Subjective norm	0.12^***^	[0.05, 0.20]	0.04	0.55	3.38		
Injunctive norm X Subjective norm	− 0.00	[− 0.03, 0.03]	0.01	− 0.01	− 0.12		

*B*,unstandardized regression coefficient; *ß*, standardized regression coefficient; CI, confidence interval; LL and UL indicate the lower and upper limits of a confidence interval, respectively. *b* represents unstandardized regression weights.

*p < 0.05. **p < 0.01. ***p < 0.001.

**Fig. 1:**
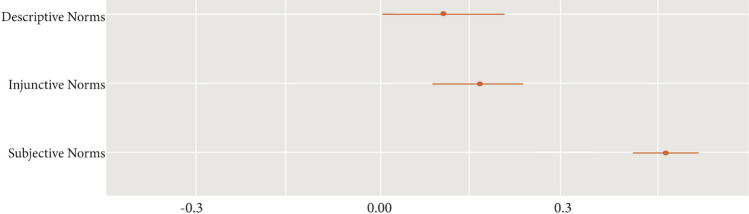
Perceived norms predicting COVID-19 vaccination intentions. *Note.* X-axis represents the standardized regression coefficients (ß)

Addressing RQ1, we examined the standardized regression coefficients for the three perceived norm constructs (see Fig. 1). We found that perceived subjective norms (*β* = 0.47) were most strongly associated with COVID-19 vaccination intentions. Perceived descriptive norms (*β* = 0.11) and injunctive norms (*β* = 0.16) had similar levels of associations with vaccination intentions.

**Fig. 2:**
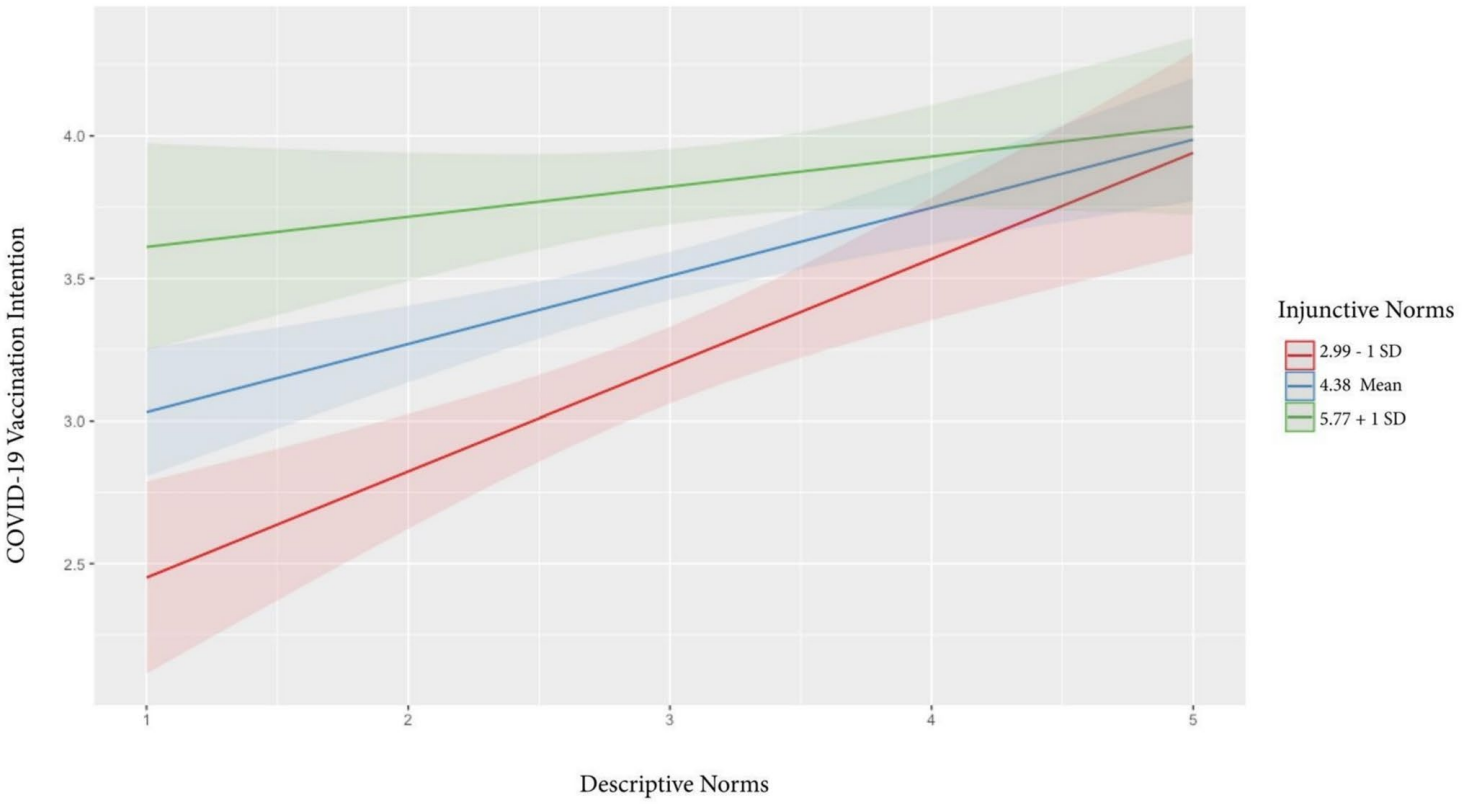
Interaction effects of descriptive and injunctive norms on vaccination intentions

**Fig. 3:**
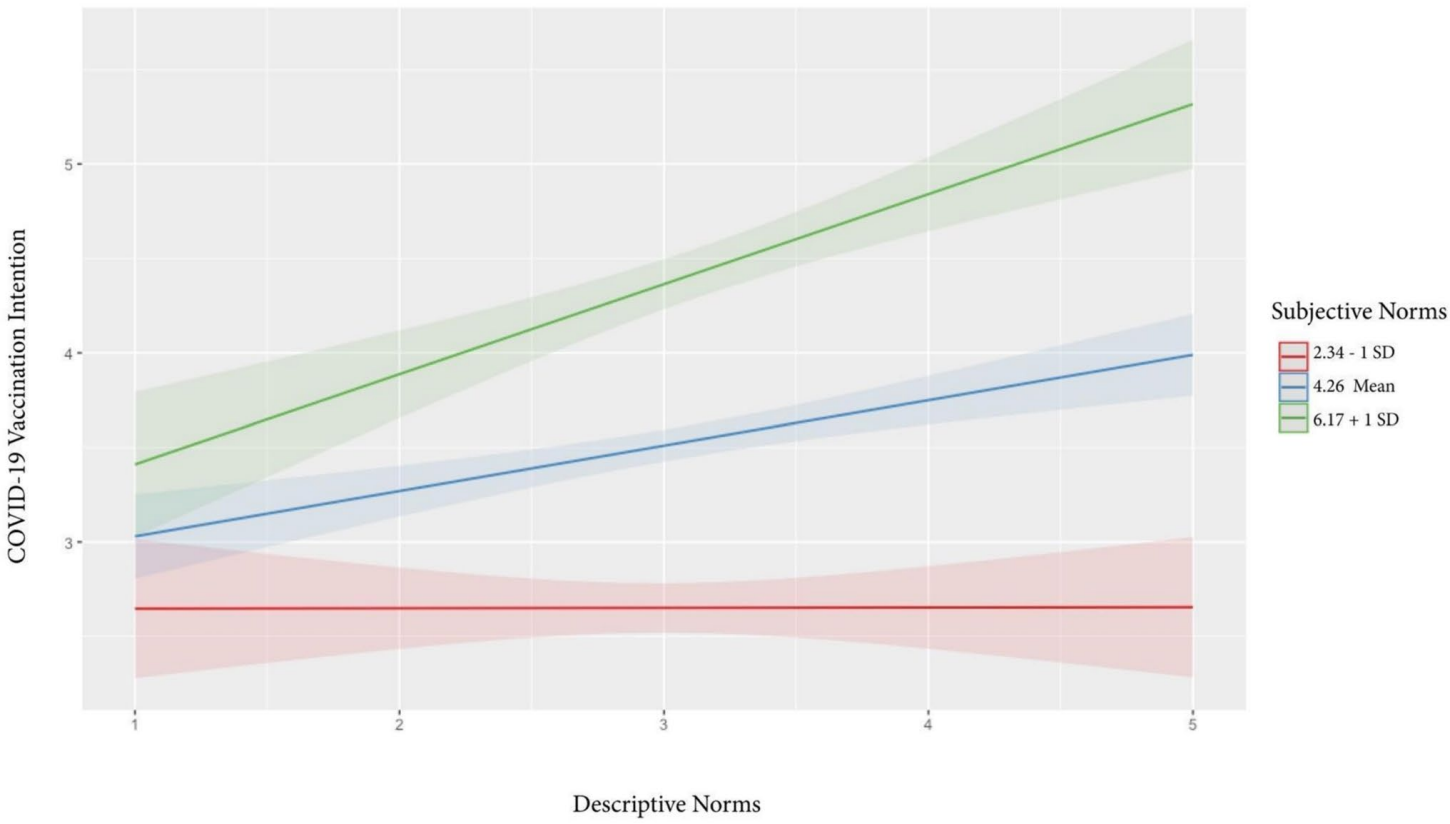
Interaction effects of descriptive and subjective norms on vaccination intentions

Addressing RQ2, the third model (Block 3 in Table 2) added the two-way interactions between descriptive norms and injunctive norms (*β* =− 0.36, *p* = 0.03), descriptive norms and subjective norms (*β* = 0.55, *p* < 0.001), and injunctive norms and subjective norms (*β* = -0.01, *p* = 0.90). We probed the interactions between descriptive norms and injunctive norms (Fig. 2) through the pick-a-point approach (Aiken & West, 1991) by using the mean of injunctive norms (4.38), mean-1SD (2.99), and mean + 1SD (5.77). The results indicated that the positive relationship between descriptive norms and vaccination intentions becomes weaker as injunctive norms increase. We also used the same technique to probe the interaction between descriptive norms and subjective norms (see Fig. 3) using the mean of subjective norms (4.26), mean-1SD (2.34), and mean + 1SD (6.17). Results indicated that the higher the subjective norms, the stronger the positive relationship between descriptive norms and vaccination intentions.

RQ3 examined the normative influence associated with Democratic and Republican reference groups among both Democratic and Republican participants. As illustrated in Table 3, unexpectedly, for Republican participants (*n* = 84), higher perceived descriptive norms among Democrats were significantly associated with greater vaccination intentions (*b* = 0.49, *p* = 0.01). As expected, higher perceived injunctive norms among Republicans were associated with greater vaccination intentions (*b* = 0.40, *p* = 0.001). Table 4 revealed that for Democratic participants (*n* = 920), higher perceived descriptive norms among Democrats were significantly associated with greater vaccination intentions (*b* = 0.31, *p* < 0.001). Moreover, higher perceived injunctive norms among Democrats were associated with greater vaccination intentions (*b* = 0.48, *p* < 0.001).

**Table 3:** Normative influence on COVID-19 vaccination intentions among Republican participants (*N* = 84)

Predictor	*b*	*b* 95% CI [LL, UL]	*ß*	Fit
*Block 1*				
Constant	2.79^***^	[1.45, 4.14]		
Age	0.02	[− 0.01, 0.06]	0.17	
Female	0.41	[− 0.56, 1.37]	0.09	
Non-binary	3.51	[− 0.51, 7.52]	0.19	
Other Gender	0.87	[− 2.04, 3.79]	0.07	
Education Level	− 0.26	[− 0.59, 0.06]	− 0.19	
				*R*^*2*^ = 0.075
				95% CI[0.00,0.15]
*Block 2*				
Constant	− 0.80	[− 2.24, 0.64]		
Age	0.01	[− 0.02, 0.03]	0.04	
Female	0.37	[− 0.38, 1.13]	0.09	
Non-binary	2.29	[− 0.91, 5.50]	0.13	
Other gender	− 0.39	[− 2.74, 1.96]	− 0.03	
Education level	− 0.17	[− 0.45, 0.10]	− 0.13	
Descriptive Democrat norms	0.49^**^	[0.12, 0.85]	0.30	
Descriptive Republican norms	0.12	[− 0.29, 0.52]	0.07	
Injunctive Democrat norms	0.07	[− 0.17, 0.32]	0.07	
Injunctive Republican norms	0.40^**^	[0.16, 0.65]	0.41	
				*R*^*2*^ = 0.472^***^
				95% CI[0.24,0.55]

*b*, unstandardized regression coefficient; *ß*, standardized regression coefficient; CI, confidence interval; LL and UL indicate the lower and upper limits of a confidence interval, respectively.

^*^ *p* < 0.05. ^**^ *p* < 0.01. ^***^ *p* < 0.001.

**Table 4:** Normative influence on COVID− 19 vaccination intentions among Democratic participants (*N* = 920)

Predictor	*b*	*b* 95% CI [LL, UL]	*ß*	Fit
*Block 1*				
Constant	2.72^**^	[2.30, 3.15]		
Age	0.02^***^	[0.01, 0.03]	0.17	
Female	− 0.42^**^	[− 0.66, − 0.17]	− 0.11	
Non-binary	− 0.32	[− 1.12, 0.48]	− 0.03	
Other Gender	0.78	[− 0.11, 1.68]	0.06	
Education Level	0.12^**^	[0.05, 0.20]	0.11	
				*R*^*2*^ = 0.055^***^
				95% CI[0.03,0.08]
*Block 2*				
Constant	0.13	[− 0.37, 0.63]		
Age	0.01^**^	[0.00, 0.02]	0.09	
Female	− 0.32^**^	[− 0.53, − 0.12]	− 0.09	
Non-binary	− 0.41	[− 1.08, 0.26]	− 0.03	
Other Gender	0.44	[− 0.31, 1.18]	0.03	
Education Level	0.05	[− 0.01, 0.12]	0.04	
Descriptive Democrat norms	0.31^***^	[0.20, 0.43]	0.18	
Descriptive Republican norms	− 0.07	[− 0.17, 0.03]	− 0.04	
Injunctive Democrat norms	0.48^***^	[0.41, 0.55]	0.45	
Injunctive Republican norms	0.01	[− 0.06, 0.08]	0.01	
				*R*^*2*^ = 0.350^***^
				95% CI[0.30,0.39]

*b*,unstandardized regression coefficient; *ß*, standardized regression coefficient; CI, confidence interval; LL and UL indicate the lower and upper limits of a confidence interval, respectively.

*p < 0.05. **p < 0.01. ***p < 0.001.

## Discussion

### Summary of findings

This study investigates how perceived social norms influence COVID-19 vaccination intentions among Black Americans (*N* = 1,497). Our findings reveal that Black Americans are more likely to get a COVID-19 vaccine when they perceive high descriptive norms (others getting vaccinated), injunctive norms (societal approval of vaccination), and subjective norms (value placed on vaccination by significant others). Moreover, perceived norms interacted to affect COVID-19 vaccination intentions. Specifically, the positive association between descriptive norms and vaccination intentions weakens as injunctive norms intensify but strengthens as subjective norms become more prominent. Our study also delineates the significant influence of political identity: Republicans are influenced by both in-group injunctive norms and out-group descriptive norms, while Democrats respond predominantly to in-group norms.

### Theoretical contributions

Our study aligns with the theory of normative social behavior (Real & Rimal, 2007; Rimal & Real, 2005) and the theory of planned behavior (Ajzen, 1991), highlighting the influential role of perceived norms on vaccination intentions among Black Americans. All three social norms – descriptive norm, injunctive norm, and subjective norm – were positively associated with Black Americans’ COVID-19 vaccination likelihood. These findings are consistent with research on the general populace (e.g., Baeza-Rivera et al., 2021; Ogilvie et al., 2021), indicating an increased propensity for vaccination when individuals observe vaccination uptake by others, societal endorsement, and recommendations from important people in their lives. Notably, subjective norms, which reflect the expectations of significant others, exert a more substantial influence on Black Americans’ vaccination intentions than either descriptive or injunctive norms, underscoring the potent impact of close social networks over broader societal influences and suggesting that different types of norms may be more or less salient in different social groups. In sum, our findings bolster the theoretical proposition that perceived norms are crucial predictors of vaccination behaviors.

Moreover, our study elucidates the complex interplay among social norms and their collective impact on vaccination intentions. Specifically, the positive association between descriptive norms and vaccination intentions diminishes as injunctive norms intensify. This could imply that the persuasive power of moral obligations can overshadow the mere observation of others’ actions in determining health behaviors with societal implications, such as vaccination. In contrast, the positive relationship between descriptive norms and vaccination intentions becomes stronger as subjective norms intensify. This finding suggests that subjective norms, which function on a personal relationship level, can act as a magnifying glass, making the actions of others more salient and persuasive. Past studies also have highlighted the interaction effect of different forms of norms. For instance, Staunton et al. (2014) found that participants exposed to a positive injunctive norm reported significantly lower intentions of healthy eating when a negative descriptive norm was made salient. Yang (2017) found that peer injunctive norms weakened the relationship between descriptive norms and intentions to consume alcohol among students with a strong interdependent self-construal but strengthened it among those with a weak interdependent self-construal. These findings, together with the current study, underscore the interrelated effects of social norms on some health behaviors. When more empirical evidence emerges, we encourage future research to build an integrated model that considers the interactions among descriptive, injunctive, and subjective norms.

Finally, our study highlights the theoretical importance of differencing in-group and out-group norms in predicting individuals’ vaccination behaviors. Among Black American Democrats, only in-group norms (perceived descriptive and injunctive norms among Democrats) influenced their vaccination intentions, while out-group norms (perceived descriptive and injunctive norms among Republicans) had no significant impact. This finding aligns with the social identity framework (Hogg & Reid, 2006), suggesting that people tend to conform to their in-group norms to derive a sense of belonging and self-esteem. Conversely, Black American Republicans show a unique pattern where their vaccination intentions are influenced by the descriptive norms of the out-group (Democrats) and the injunctive norms from their own group (Republicans). This could reflect a more complex social identity dynamic among Black American Republicans, where they are open to behavioral cues from Democrats but rely on their own group for moral guidance and approval regarding vaccination. Another possible explanation is the numerical rarity of Republican identification among Black Americans (Pew Research Center, 2024), which may limit the visibility and salience of in-group behaviors and increase reliance on the more prominent majority-group norms from Democrats. The small sample size of Republicans may also introduce sampling errors that could influence the results. And we also cannot say whether the same patterns influenced by partisan identity would hold among other racial groups. Future research should explore how partisan identity among a range of racial groups interacts with perceived social norms to influence COVID-19 and other vaccination intentions.

### Practical implications

Our findings offer valuable insights for communicators and public health professionals in promoting COVID-19 vaccination. The significant association between descriptive, injunctive, and subjective norms with vaccination intentions suggests a nuanced approach by public health campaigns to boost vaccine uptake. Campaigns should highlight the widespread acceptance of vaccines (descriptive norms), feature endorsements from esteemed groups or individuals (injunctive norms), and emphasize personal recommendations from friends and family (subjective norms). For example, in the study by Santos et al. (2021), a descriptive norm message conveyed to healthcare workers that a significant proportion of providers (“80%” was included in the intervention message) and over 11 million Americans, including many of their colleagues, had already received vaccinations, leading to a significant rise in vaccination rates. Our finding that the influence of descriptive norms diminishes in the face of strong injunctive norms indicates that campaigns need to address and potentially counteract negative injunctive norms, ensuring influential community figures or groups visibly support vaccination. Again, this finding is supported by prior empirical work. For example, Ryoo and Kim (2021) tested COVID-19 vaccine messages that combined both injunctive and descriptive norms, emphasizing the percentage of available vaccination appointments and the shared social responsibility to get vaccinated. They found that participants exposed to this combined normative message showed higher vaccine acceptance compared to those who received only a descriptive norm message. Furthermore, the finding that subjective norms can amplify the impact of descriptive norms suggests that by combining the evidence of widespread vaccination with personal endorsements from friends, family, and peers, health messages can become more compelling. The particularly pronounced effect of subjective norms underscores the potential effectiveness of grassroots-level vaccination campaigns. Such initiatives could involve mobilizing community leaders, local influencers, family members, or friends to vocalize their support for COVID-19 vaccination.

Furthermore, the influence of political affiliations on vaccination intentions suggests that health campaigns should be customized to resonate with the values of different political groups. Recognizing the stronger in-group influence suggests that tailored messaging, which speaks directly to each party’s values and concerns, could be more effective. For instance, messages targeting Republicans might emphasize personal freedom and individual responsibility, while messages for Democrats could focus on community well-being and collective responsibility (Chung & Kim, 2023). In addition, given the cross-party influence observed among Black American Republicans, health campaigns might consider tailoring messages that demonstrate broad, bipartisan support for vaccination, showing that members of both parties are getting vaccinated.

### Limitations

This study has limitations that should be considered when interpreting the results. First, the sample had a predominant representation of Democrats, resulting in an underrepresentation of Republicans. This partisan imbalance limits the generalizability of our findings to Republican populations, who may respond differently to vaccination messaging and social norms given known differences in political orientation and vaccine attitudes. Future studies should aim for a more politically diverse sample to reassess our key questions. Second, our study focused on perceived norms within the two major political party reference groups, Democrats and Republicans. We were not able to analyze participants who identified as Independents or members of other political parties separately because our measures did not include normative reference items specific to these groups. Future research should explore how these under-examined groups respond to normative influences, as they represent important segments of the population. Third, our study focused on vaccination intentions rather than actual vaccine uptake. While intentions provide a useful proxy for potential behavior, they do not guarantee follow-through. Measuring actual vaccination rates would provide a more accurate picture of how social norms influence health behaviors. Future research that can track real-world vaccine adoption or access vaccine records would allow a closer examination of how intentions translate into action. Finally, the cross-sectional design of this study precludes causal inferences. While our findings suggest associations between social norms and vaccine intentions, we cannot determine causality due to the single time point measurement. Longitudinal studies or randomized field experiments are recommended to assess the causal impact of social norms on health behaviors, providing a more rigorous test of the theories underlying normative influence on vaccine behavior.

## Conclusion

In conclusion, our study underscores the importance of crafting public health messages that are finely tuned to specific social dynamics and identities within the Black American community. Given the strong association of subjective norms with vaccination intentions, leveraging personal endorsements from trusted community figures, family, and friends may be particularly effective. The complex interplay between descriptive and injunctive norms suggests that campaigns should carefully balance messages about social acceptance and behavior prevalence. Additionally, recognizing the unique partisan dynamics—where Republicans within this demographic respond to both in-group moral cues and out-group behaviors—suggests that bipartisan support and messaging that resonates across political divides may be especially impactful in encouraging vaccine uptake.

## Data Availability

The data for this project are available upon request from the corresponding author.

## Footnotes

1 A functional food has extra benefits, provided by adding new or more of its existing ingredients (Nystrand & Olsen, 2020).
